# Molecular subtypes of breast cancer and amplification of topoisomerase II*α*: predictive role in dose intensive adjuvant chemotherapy

**DOI:** 10.1038/sj.bjc.6603449

**Published:** 2006-10-31

**Authors:** J Hannemann, P Kristel, H van Tinteren, M Bontenbal, Q G C M van Hoesel, W M Smit, M A Nooij, E E Voest, E van der Wall, P Hupperets, E G E de Vries, S Rodenhuis, M J van de Vijver

**Affiliations:** 1 Division of Experimental Therapy, The Netherlands Cancer Institute, Plesmanlaan 121, 1066 CX Amsterdam, The Netherlands; 2 Division of Diagnostic Oncology, The Netherlands Cancer Institute, Plesmanlaan 121, 1066 CX Amsterdam, The Netherlands; 3 Biometrics Department, The Netherlands Cancer Institute, Plesmanlaan 121, 1066 CX Amsterdam, The Netherlands; 4 Department of Medical Oncology, The Erasmus Medical Center/Daniel den Hoed Cancer Center, Postbus 5201, 3008 AE Rotterdam, The Netherlands; 5 Department of Medical Oncology, University Medical Center St Radboud, Postbus 9101, 6500 HB Nijmegen, The Netherlands; 6 Department of Medical Oncology, Medical Hospital Twente, Postbus 50.000, 7500 KA Enschede, The Netherlands; 7 Department of Medical Oncology, University Medical Center Leiden, Postbus 9600, 2300 RC Leiden, The Netherlands; 8 Department of Medical Oncology, University Medical Center Utrecht, Postbus 85500, 3508 GA Utrecht, The Netherlands; 9 Department of Medical Oncology, Free University Hospital Amsterdam, Amsterdam, The Netherlands; 10 Department of Medical Oncology, University Hospital Maastricht, Postbus 5800, 6202 AZ Maastricht, The Netherlands; 11 Department of Medical Oncology, University Medical Center Groningen, Postbus 30.001, 9700 RB Groningen, The Netherlands; 12 Division of Medical Oncology, The Netherlands Cancer Institute, Plesmanlaan 121, 1066 CX Amsterdam, The Netherlands

**Keywords:** breast cancer, topoisomerase II*α*, molecular subtypes, chemotherapy, sensitivity

## Abstract

Benefit from chemotherapy treatment in breast cancer patients is determined by the molecular make-up of the tumour. In a retrospective analysis, we determined the molecular subtypes of breast cancer originally defined by expression microarrays by immunohistochemistry in tumours of patients who took part in a randomised study of adjuvant high-dose chemotherapy in breast cancer. In addition, the topoisomerase II*α* (TOP2A) amplification status was determined by fluorescence *in situ* hybridisation and chromogenic *in situ* hybridisation. 411 of the 753 tumours (55%) were classified as luminal-like, 137 (18%) as basal-like and 205 (27%) as human epithelial receptor type 2 (*HER2*) amplified. The basal-like tumours were defined as having no expression of ER and HER2; 98 of them did express epidermal growth factor receptor and/or cytokeratin 5/6. The luminal-like tumours had a significantly better recurrence free and overall survival than the other two groups. From the 194 HER2-positive tumours, 47 (24%) were shown to harbour an amplification of *TOP2A*. Patients with an *HER2*-amplified tumour randomised to the high-dose therapy arm did worse than those in the conventional treatment arm, possibly caused by the lower cumulative anthracycline dose in the high-dose arm. The tumours with a *TOP2A* amplification contributed hardly to this difference, suggesting that *TOP2A* amplification is not the cause of the steep dose–response curve for anthracyclines in breast cancer. Possibly, the difference of the cumulative dose of only 25% between the treatment arms was insufficient to yield a survival difference.

The development of breast cancer is associated with a number of genetic alterations involving the inactivation of tumour suppressor genes and the activation of oncogenes. A dominant mechanism leading to oncogene activation is the amplification of specific genomic regions. Human epithelial receptor type 2 (HER2) (also known as c-erb-B2) is frequently amplified in breast cancer and its overexpression is associated with poor clinical outcome (Slamon *et al*, 1987; Press *et al*, 1997). Amplification of HER2 can be detected in about 15–30% of invasive breast cancers and these carcinomas are often characterised by poor histologic grade, high numbers of proliferating cells, DNA aneuploidy and the lack of expression of oestrogen and progesterone receptors (Ross and Fletcher, 1998). In addition, HER2 status has been suggested to alter the chemosensitivity of cancer cells: HER2 overexpressing cells have been reported to be relatively resistant to endocrine therapy and marked differences may exist between HER2-positive and -negative tumours concerning their response to alkylating agents and to anthracyclines (Muss *et al*, 1994).

The *HER2* amplicon is located on chromosome 17q12–21 that harbours other biologically interesting genes such as topoisomerase II*α* (*TOP2A*), which encodes for an important enzyme in DNA replication and in cell cycle progression. It catalyses the ATP-dependent transport of one DNA double helix through another by the transient introduction of a DNA double-strand break (Wang, 2002). This protein is also the target enzyme for anthracyclines, which are among the most effective chemotherapeutic agents in breast cancer. It has been suggested that amplification of *TOP2A* and not overexpression of HER2 may, in fact, be the predictive marker for response to anthracyclines (Coon *et al*, 2002; Di Leo *et al*, 2002). This notion has been strengthened by a subgroup analysis of the BCIRG-006 study, suggesting that the combination of trastuzumab/doxorubicin is only superior to trastuzumab/docetaxel in tumours harbouring an amplification of *TOP2A* (Press *et al*, 2005).

Gene expression profiling has identified several subtypes of invasive breast carcinomas associated with different clinical outcomes (Sorlie *et al*, 2001; Sorlie *et al*, 2003). Perou *et al* (2000) and Sorlie *et al* (2003) were able to distinguish different groups of breast cancer based on differences in gene expression: one group was called erb-B2-like and is characterised by HER2 overexpression, there were several subgroups called luminal A, B and normal-like that all show expression of the oestrogen receptor (ER) but no HER2 amplification, and one called the ‘basal-like’, which does not express HER2 or hormone receptors but cytokeratin 5/6 (CK5/6) and/or epidermal growth factor receptor (EGFR). It was suggested that patients with basal-like tumours would have a very poor prognosis. Obviously, neither trastuzumab nor endocrine treatments are effective in this group. Additional studies using immunohistochemistry (IHC) have identified a pattern of four antibodies that is able to distinguish these subgroups in archival paraffin-embedded tissue (van de Rijn *et al*, 2002).

Recently, the large Dutch National Study investigating the effect of high-dose alkylating chemotherapy in high-risk breast cancer was published (Rodenhuis *et al*, 2003). This study concluded that a benefit of high-dose chemotherapy is confined to patients with HER2-negative tumours, whereas HER2-positive tumours responded better to conventional, anthracycline-based chemotherapy. These results were subsequently confirmed in additional analyses with a longer median follow-up (87 months) (Rodenhuis *et al*, 2006).

In tumours of patients participating in this study, we retrospectively analysed the potential differences in prognosis or treatment effect in the molecular subtypes of breast cancer as defined by IHC. We also investigated whether amplification status of *TOP2A* is correlated with specific benefit from conventional-dose anthracycline-based chemotherapy compared to high-dose alkylating chemotherapy in patients with HER2-positive tumours.

## MATERIALS AND METHODS

### Patients

A total of 885 breast cancer patients were enroled into a multicentre phase III trial. This study investigated the benefit of high-dose adjuvant chemotherapy in patients with four or more axillary lymph node metastases. The design of the study has been described previously (Rodenhuis *et al*, 2003). Briefly, patients were included when they were younger than 56 years of age, had undergone surgery for their primary breast cancer and had at least four tumour-positive axillary lymph nodes but no distant metastases.

Patients were randomised to either five courses of anthracycline-based conventional-dose chemotherapy or to the high-dose group, where the fifth course of conventional-dose chemotherapy was replaced by one course of high-dose chemotherapy (cyclophophamide, thiotepa and carboplatin) followed by autologous peripheral blood progenitor-cell transplantation.

Of 753 of the 885 patients, paraffin blocks of the primary tumour could be procured. All tumour specimens were reviewed. Additional IHC and chromogenic *in situ* hybridisation (CISH) or fluorescence *in situ* hybridisation (FISH) stainings were performed.

### Chromogenic *in situ* hybridisation

Chromogenic *in situ* hybridisation was performed using the Spot-Light CISH Polymer Detection Kit (Zymed, San Francisco, CA, USA). The HER2 DNA probe and the TOP2A Amplification probe were also received from Zymed (San Francisco, CA, USA). Experiments were performed according to the manufacturer's protocol, in brief as follows:

Paraffin-embedded tissue specimens were immersed in xylene twice and then soaked in 100% ethanol for three times. Heat pretreatment was performed in a microwave step by using Zymed's pretreatment solution, DNA retrieval was carried out by a pepsin step at room temperature. The slides were dehydrated in graded ethanol series and the probe was applied to the tissue specimen and denatured by a 5 min step at 95°C. Slides were incubated at 37°C and washed the next day. Immunodetection was performed by DAB-chromogen. Before scoring, tissue specimens were counterstained with hematoxylin.

### Fluorescence *in situ* hybridisation

The TOP2A FISH pharmDx™ Kit (DAKO, Glostrup, Denmark) was used to determine amplification of TOP2A by FISH, according to the manufacturer's protocol. Briefly, the tissue specimens were deparaffinised by baking and rehydrated by graded ethanol series. The DNA target was retrieved by pepsin treatment, the probe was applied to the specimen and both were co-denatured at 82°C for 5 min. Hybridisation took place at 45°C over night. The tissue specimens were washed the next day and were then dehydrated by graded ethanol series. Fluorescence signals were counted and *TOP2A* amplification was determined.

### Immunohistochemistry

Microscopic slides containing 5-*μ*m sections of the tumour material were stained with antibodies, which are commercially available. The ER was stained with a mouse monoclonal antibody (clone 1D5+6F11, NeoMarkers, Fremont, CA, USA) in a dilution of 1 : 50 with an autoclave antigen retrieval in citrate buffer (pH 6.0). The CK5/6 antibody (clone D5/16 B4, DakoCytomation, Glostrup, Denmark) was diluted 1 : 200. Antigen retrieval was in the autoclave in citrate buffer (pH 6.0). The HER2 antibody (clone 3B5) (van de Vijver *et al*, 1988) was diluted 1 : 80 000 with a 15-min microwave antigen retrieval in citrate buffer. For staining the EGFR, samples were treated with 0.1% Pronase E (Sigma-Aldrich, St Louis, MO, USA) for 20 min at room temperature and stained with EGFR antibody (clone 111.6, NeoMarkers, Fremont, CA, USA) in a dilution of 1 : 100. Detection was performed with PowerVision+ (ImmunoVision Technology, Brisbane, CA, USA) using an HRP-conjugated second antibody.

### Scoring

Samples were scored as ER positive by IHC, when at least 10% of the tumour cells showed staining of the ER in the nuclei. Cytokeratin 5/6 and EGFR were reported as positive if membrane staining for EGFR and any membrane or cytoplasmic staining for CK5/6 could be observed. A sample was considered to be HER2 positive when either a strong membrane staining (3+) could be observed by IHC or CISH revealed amplification of *HER2* in samples with weak (1+ or 2+) membrane staining at IHC.

The assessment of the gene status by CISH was as follows: at least 30 tumour cells were evaluated. According to the manufacturer's instructions, tumours with an average of less than five spots per nucleus were considered to be *HER2* non-amplified, samples with an average of at least or more than five spots per nucleus were considered to be *HER2* amplified. The same scoring system was used for defining *TOP2A* gene amplification.

For tumour material examined by FISH, at least 40 nuclei were evaluated and tumours were considered to be amplified for *HER2* or *TOP2A*, respectively, when a probe/centromer ratio higher than or equal to two was observed. Samples with a ratio of smaller than two were scored as not amplified and a ratio ⩽0.5 was defined as deletion.

During the course of our study, CISH emerged as an equivalent alternative to FISH. Therefore, cases tested in the early phase of the study were analysed by FISH, whereas samples tested in the later phase of the study were assessed by CISH.

### Statistical analysis

The association of (a) the amplification of *TOP2A* and (b) the molecular subtypes of breast cancer with recurrence-free and overall survival was analysed using the Kaplan–Meier technique and log-rank tests to compare different groups with each other and Cox-proportional hazard analysis for calculating hazard ratios with 95% confidence intervals. Recurrence-free survival time was calculated from date of randomisation until time of first event, either recurrence of disease or death. Overall survival was defined as time from randomisation until date of death or date of last follow-up. The interaction of the parameters with treatment in relation to outcome was depicted by means of a Forest-plot. In this Forest-plot, the closed squares represent the point estimate of the effect of high-dose chemotherapy (HD) *vs* conventional-dose therapy (CONV) within that particular subgroup (with the size of the square corresponding to its variance) and the lines correspond to the 99% confidence interval of that effect. The open figure at the bottom shows the overall effect of high-dose chemotherapy *vs* CONV for this particular cohort of patients.

## RESULTS

Between August 1993 and July 1999, 885 patients from 10 Dutch hospitals were randomised between conventional-dose and high-dose chemotherapy. Patients in the conventional arm received five courses of anthracycline-based chemotherapy, whereas patients in the high-dose arm received only four courses (Rodenhuis *et al*, 2003). We identified the molecular subtypes of breast cancer in 753 out of 885 patients by the combination of four immunohistochemical markers (HER2-positive, luminal ER-positive and basal-like CK5/6, and/or EGFR-positive breast cancer). The *TOP2A* amplification status was assessed by CISH or FISH in the subgroup of HER2-positive tumours (*n*=194).

### Patient and tumour characteristics: HER2-positive patients with TOP2A data available

A total of 194 patients were analysed in this subgroup of HER2-positive tumours. Ninety-four of these had been randomly treated in the high-dose chemotherapy arm, 100 had been treated in the conventional-dose chemotherapy arm. The two groups had similar clinical characteristics (Table 1).

### Patient and tumour characteristics: breast cancer subtype analyses

The analysis of the four different subtypes of breast cancer was performed on samples of 753 patients. A total 376 patients were randomised to the high-dose chemotherapy arm and 377 patients to the conventional-dose treatment arm. There was no difference in clinical characteristics between the patients from whom paraffin-embedded material was available and those from whom samples were not available (Table 2).

### Correlation of *HER2* and *TOP2A* amplification by CISH and FISH

We performed FISH experiments to detect *TOP2A* amplification on all samples that were scored as HER2 1+, 2+ or 3+ by IHC and from which paraffin-embedded tumour material was available. As *TOP2A* gene amplification is extremely rare (and not detected at all in some studies), we did not test immunohistochemically HER2-negative tumours for *TOP2A* gene amplification.

Of the 211 samples, an interpretable FISH-TOP2A result was obtained in 203 cases. On a further 16 samples, we performed CISH-TOP2A staining and 10 of these were appropriate for scoring. In total, we could establish the amplification status of *TOP2A* in 213 cases. In total, *TOP2A* data are available from 194 HER2-positive and 19 HER2-negative tumours. Forty-seven of the samples (22%) showed co-amplification of *TOP2A* and *HER2*, whereas the majority of the cases were positive for HER2, but had no amplification of *TOP2A* (69%). Among the cases without *TOP2A* gene amplification (but with *HER2* gene amplification), there were nine cases with loss of one *TOP2A* allele. The results are summarised in Table 3.

### Relation of TOP2A amplification with survival

It has been suggested that sensitivity to anthracycline-based chemotherapy is associated with amplification and overexpression of TOP2A (Coon *et al*, 2002; Di Leo *et al*, 2002). We therefore examined the presence of a (recurrence-free) survival advantage for patients with *TOP2A*-amplified tumours in the conventional-dose arm over those in the high-dose arm that contained a 20% lower cumulative dose of anthracyclines.

**Figure 1:**
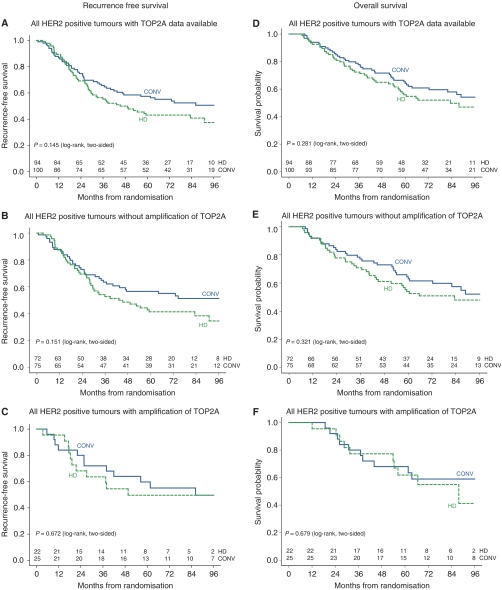
Recurrence-free and overall survival among the HER2-positive patients. (**A** and **D**) all patients with TOP2A data; (**B** and **E**) 147 patients without amplification of TOP2A; (**C** and **F**) 47 patients with amplification of TOP2A. Tumours were considered to be TOP2A amplified, when there was a probe/centromer ratio ⩾2 by FISH or more than fish spots detected by CISH.

A total of 194 patients, from whom information on *TOP2A* gene amplification, chemotherapy treatment and clinical follow-up were available, had tumours with *HER2* gene amplification. We separated the patients with and without amplification of the *TOP2A* gene in two groups and generated Kaplan–Meier survival curves with a median follow-up of 87 months. For HER2-positive patients with *TOP2A* amplification, no survival differences between high-dose and conventional chemotherapy seems to be present (Figure 1). The curves do not suggest an apparent interaction between or confounding effect of TOP2A on the relation between treatment and survival.

Also the subgroups analysis of HER2-positive/*TOP2A*-negative *vs* HER2-positive/*TOP2A*-positive samples did not show an effect of TOP2A amplification (data not shown). Additionally, we analysed the impact of *TOP2A* amplification status on treatment-related survival in the group of HER2 positive, ER-negative patients separately to exclude any indirect endocrine effect. The results are not different from the results of the analyses of the whole set of cases (data not shown), although it should be kept in mind that these subsets are relatively small and therefore confidence intervals are wide.

### Determination of the different subtypes of invasive breast cancer by IHC

Several subtypes of breast cancer have been identified recently and have been correlated with clinical outcome (Sorlie *et al*, 2001, 2003; van de Rijn *et al*, 2002). These results have been translated to classical IHC (Nielsen *et al*, 2004). We therefore used the combination of four antibodies to identify these subtypes within our patient cohort and correlated these results with survival after high-dose or conventional chemotherapy.

We first stained all cases from which paraffin-embedded material was available with antibodies against HER2, ER, CK5/6 and EGFR. Twenty-five percent of the samples were positive for HER2, 70% were positive for ER, 12% of the cases showed strong expression of CK5/6 and 15% of the cases expressed EGFR. Table 4 summarises the frequencies of positive and negative staining of these four markers. We also investigated the focal staining of CK5/6. From 92 patients stained positive for CK5/6, 28 showed focal staining ⩽5%, 28 samples showed staining between 5 and 50% and in 36 cases at least 50% of the tumour cells were positive for CK5/6.

Subsequently, we divided 753 samples into three breast cancer subtypes according to the results obtained from the IHC and the additional CISH staining. How the different molecular subtypes were defined by IHC is shown in Table 5. A total of 205 cases (27%) were assigned to the HER2-positive group and 411 samples (55%) to the luminal, ER-positive group. Thirteen percent of the samples showed expression of CK5/6 and/or EGFR and are therefore termed basal-like. Five percent of the cases do not show expression of the evaluated markers (Table 5). For analytic purposes, the tumours are categorised as part of the basal-like group. In the HER2-positive group, 15% of the cases also expressed CK5/6 or EGFR. In the ER-positive samples, 6% showed expression of CK5/6 or EGFR. Most of the tumours with amplification of TOP2A show neither expression of CK5/6 nor EGFR, only in two TOP2A-amplified samples expression of CK5/6 or EGFR could be detected.

### Association of the breast cancer subtypes with patient's survival

**Figure 2:**
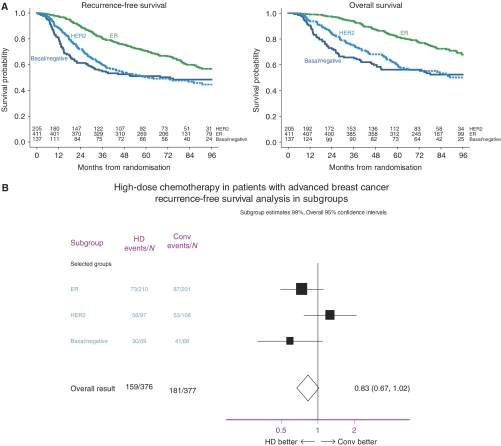
(**A**) Recurrence-free and overall survival among the HER2-expressing, ER-expressing and the basal-like subtype of invasive breast cancer. (**B**) Forrest plot – Correlation of the recurrence free survival in these subgroups with response to conventional anthracycline-based or alkylating high-dose chemotherapy, respectively.

It has been reported that the basal type of breast cancer is correlated with poor clinical outcome (van de Rijn *et al*, 2002). We therefore used the patient cohort of 753 patients that could be grouped into three subtypes of invasive breast cancer and studied the recurrence-free and overall survival of these groups. Kaplan–Meier survival curves of the ER expressing, the HER2 expressing and the basal-like subgroup are shown in Figure 2A. Patients with luminal ER-expressing tumours showed better survival compared to those in the other two groups (5-year overall survival 81%, 95% confidence interval 78–85%). If one compares the basal-like tumours and the HER2-amplified ones, a similar recurrence-free and overall-survival probability is observed (5-year overall survival rates 56 and 60%, 95% confidence intervals 48–65 and 54–67%, respectively). Importantly, the patients with HER2-positive tumours did not receive adjuvant trastuzumab in this study.

For the three different subgroups, we also determined the hazard ratios of recurrence-free survival after conventional-dose or high-dose chemotherapy, respectively (Figure 2B). Oestrogen receptor-positive tumours and tumours in the basal-like/negative subgroup seem to derive benefit from high-dose chemotherapy, whereas a trend is seen that tumours with HER2 amplification might benefit more from conventional chemotherapy. This finding is in line with previously published results (Rodenhuis *et al*, 2003; Rodenhuis *et al*, 2006)

## DISCUSSION

In this study, the pathology and survival data of a large cohort of patients with high-risk primary breast cancer were reanalysed (Rodenhuis *et al*, 2003; Rodenhuis *et al*, 2006) We retrospectively studied the association of the molecular subtyping of breast cancer based on IHC with treatment outcome and we investigated a possible influence of the anthracycline dose on treatment effect for tumours with a *TOP2A* amplification.

Several studies have suggested that anthracyclines such as doxorubicin have a steep dose–response curve in HER2-positive breast cancer (Muss *et al*, 1994), but a supposed sensitivity to anthracyclines has not been confirmed invariably (Paik *et al*, 1998; Thor *et al*, 1998; Vincent-Salomon *et al*, 2000). As the anthracyclines target the enzyme TOP2A, it has been suggested that co-amplification of *TOP2A* could be the actual mechanism underlying the relationship between HER2 overexpression and the efficacy of optimally dosed anthracycline-based chemotherapy. This concept is also supported by the finding that cells with amplification of both *HER2* and *TOP2A* are highly sensitive to anthracyclines *in vitro* (Jarvinen *et al*, 2000). In the clinical setting, only a few studies have been published regarding the predictive value of *TOP2A* amplification in breast cancer patients (Coon *et al*, 2002; Di Leo *et al*, 2002). A recent study by Knoop *et al*, 2005 investigated TOP2A changes in 773 tumour samples. They found that amplification (and possibly deletion) of the *TOP2A* gene seem to be predictive for the effect of adjuvant epirubicin containing therapy. If it could be unambiguously shown that the difference in sensitivity to anthracyclines between HER2-positive and HER2-negative tumours is explained by the amplification of the *TOP2A* gene, clinical decision making in adjuvant therapy should, in part, be guided by assessing the *TOP2A* gene amplification status. As *TOP2A* gene amplification is only found in HER2-amplified tumours, a sensible testing algorithm would be to test only HER2-amplified cases for *TOP2A* status.

In the study presented here, we determined the amplification status of *TOP2A* in all HER2-positive tumours of the patient cohort of the Dutch National trial of high-dose chemotherapy (Rodenhuis *et al*, 2003). Among the tumours with *HER2* gene amplification, only 22% showed co-amplification of the *TOP2A* gene and 1% of the cases showed deletion of the *TOP2A* gene. These data are in agreement with the hypothesis that amplification or deletion of the *TOP2A* gene is associated with *HER2* gene amplification as a result of the colocalisation of both genes on the chromosomal region 17q12–q21. It has been suggested previously that, after initial amplification of *HER2*, additional chromosomal rearrangement events lead to either telomeric high level amplifications or interstitial deletions, which may encompass the *TOP2A* gene (Jarvinen *et al*, 1999).

We subsequently determined the survival-rates for the HER2-positive tumours of this patient cohort after conventional-dose and high-dose chemotherapy dependent on the *TOP2A* gene amplification status of the tumours. There was no relative difference in recurrence-free or overall survival of *TOP2A* gene amplified *vs* non-amplified tumours depending on the arm of the study (Figure 1). Petit *et al*, 2001 have shown that FE_100_C was more effective than FE_50_C in the neoadjuvant setting in patients with HER2-positive disease, whereas for HER2-negative disease no difference between these two schedules could be observed. Although this and other studies suggest that the efficacy of anthracyclines in HER2-positive tumours is dose dependent in the adjuvant (Muss *et al*, 1994) and neoadjuvant setting (Petit *et al*, 2001), it is possible that the difference of the cumulative dose of only 25% between the treatment arms was insufficient to yield a survival difference. Nevertheless, *TOP2A* gene amplification does not appear to be the main reason that HER2-positive tumours did worse after high-dose alkylating chemotherapy.

Based on gene expression profiling studies (Sorlie *et al*, 2001; Sorlie *et al*, 2003), invasive breast cancer can be divided into several subtypes: the basal-like, characterised by the expression of the CK5/6 and or EGFR, the luminal ER-positive groups, and a HER2 overexpressing group. This classification has been translated to classical IHC (Nielsen *et al*, 2004) and a combination of four immunohistochemical markers (ER, HER2, CK5/6 and EGFR) can be used to distinguish between these subgroups. The basal-like tumours are defined as being negative for HER2 and ER. For technical reasons, it is important to base classification not only on negative markers, as technical failure could not be detected in such cases. Therefore, we used the expression of CK5/6 and EGFR to identify basal-like tumours. Other studies also describe a relationship between the expression of c-KIT and CK17 and the basal-like subtype of breast cancer, but these markers are not that frequently positive as CK5/6 (Nielsen *et al*, 2004). The definition of the molecular subgroups based on IHC is given in Table 3. The basal-like subtype has been reported to be associated with very poor prognosis. Clearly, there may also be a difference in response to specific chemotherapeutic regimens for each of these subgroups, which would establish a predictive value in addition to the prognostic one. Within the set of patients analysed, we identified 13% of the cases as belonging to the basal-like subgroup based on the expression of CK5/6 and/of EGFR. This result confirms that of Nielsen *et al* (2004), who found that 15% of 663 invasive breast carcinomas were basal-like based on IHC and expression of either CK5/6 or HER1.

In accordance with previously published data (Rodenhuis *et al*, 2003), patients with a HER2-negative tumour appeared to benefit from high-dose chemotherapy. On the contrary, patients with HER2-positive tumours actually did better with conventional-dose anthracycline-based chemotherapy. The recurrence-free and overall survivals of the basal-like/negative group are comparable to those of the group of HER2 overexpressing tumours (Figure 2). Both groups, however, have a poor prognosis in comparison with the luminal, ER overexpressing subgroup. In a recent report, Nielsen *et al* (2004) have characterised the basal-like subtype by IHC and have studied the clinical outcome. The expression of CK5/6 or EGFR (termed HER1 in his study) was used as the hallmark of the basal-like subtype of invasive breast cancer and was indeed associated with poor survival. Similarly, van de Rijn *et al* (2002) could correlate the expression of CK5/6 in tissue arrays with poor prognosis). As the expression of CK5/6 is usually very focal within a tumour, the interpretation of tissue arrays may underestimate the percentage of CK5/6 expressing tumours. We therefore used whole tissue sections to determine the expression of CK5/6 and EGFR. In our data set, from 92 patients stained positive for CK5/6, about one-third showed focal staining ⩽5%, one-third showed staining between 5 and 50% and in about one-third of the cases at least 50% of the tumour cells were positive for CK5/6.

Of the 98 patients in the basal-like group, 45 co-expressed CK5/6 and EGFR, and 28 basal-like tumours expressed EGFR but not CK5/6. Epidermal growth factor receptor belongs to the family of tyrosine-receptor kinases and is a target for small molecule inhibitors such as erlotinib and gefitinib and for therapeutic antibodies such as cetuximab (Esteva, 2004). As these drugs are target-specific, their combination with other targeted agents or with chemotherapy could, in theory, improve outcome when EGFR overexpression is present in a basal-like tumour.

In conclusion, by employing IHC, breast cancer can conveniently be divided in at least three molecular subtypes, which have a different prognosis and which require different treatment. Although we could confirm that the *TOP2A* gene is amplified in a proportion of tumours harbouring an HER2 amplification, this abnormality does not appear to be responsible for the lack of efficacy of high-dose alkylating chemotherapy in this patient group. The existence of a (cumulative) dose–response effect of epirubicin in these patients could, however, not be excluded on the basis of our data.

Patients with tumours that were either HER2 positive or negative for the investigated markers had a significantly worse relapse-free survival than tumours of the luminal type. If adjuvant trastuzumab had been used in this study, the results in the HER2-positive tumours could have been expected to be far better. Thus, the basal-like breast cancer subtype remains the least favourable one, for which at present conventional chemotherapy rather than treatment with targeted agents remains the primary systemic treatment modality.

## Figures and Tables

**Table 1:** Clinical characteristics TOP2A samples

	**No of patients without amplification of TOP2A *N*=147 (%)**	**No of patients with amplification of TOP2A *N*=47 (%)**	**Total *N*=194 (%)**
*Treatment*			
Conventional arm	75 (51%)	25 (53%)	100 (52%)
High-dose arm	72 (49%)	22 (47%)	94 (48%)
			
*Age*			
<40 years	46 (31%)	19 (40%)	65 (34%)
40–50 years	70 (48%)	18 (38%)	88 (45%)
50+ years	31 (21%)	10 (21%)	41 (21%)
			
*Menopausal status at time of randomisation*			
Premenopausal	123 (84%)	41 (87%)	164 (85%)
Postmenopausal	13 (9%)	5 (11%)	18 (9%)
Uncertain	11 (7%)	1 (2%)	12 (6%)
			
*Type of surgery*			
Mastectomy	112 (76%)	37 (79%)	149 (77%)
Breast conserving	35 (24%)	10 (21%)	45 (23%)
			
*Tumour classification*			
T1	33 (22%)	6 (13%)	39 (20%)
T2	88 (60%)	32 (68%)	120 (62%)
T3	26 (18%)	9 (19%)	35 (18%)
			
*Number of positive lymph nodes*			
4–9	96 (65%)	28 (60%)	124 (64%)
⩾10	51 (35%)	19 (40%)	70 (36%)
			
*Grade*			
I	7 (5%)	3 (6%)	10 (5%)
II	37 (25%)	16 (34%)	53 (27%)
III	99 (67%)	26 (55%)	125 (64%)
Not determined	4 (3%)	2 (4%)	6 (3%)

TOP2A=topoisomerase II*α*.

**Table 2:** Clinical characteristics all samples

	**No of patients without available samples *N*=132**	**No of patients with formalin-fixed samples *N*=753**	**Total study population *N*=885**
*Treatment*			
Conventional arm	66 (50%)	377 (50%)	443 (50%)
High-dose arm	66 (50%)	376 (50%)	442 (50%)
			
*Age*			
<40 years	30 (23%)	195 (26%)	225 (25%)
40–50 years	74 (56%)	391 (52%)	465 (53%)
50+ years	28 (21%)	167 (22%)	195 (22%)
			
*Menopausal status at time of randomisation*			
Premenopausal	103 (78%)	630 (84%)	733 (83%)
Postmenopausal	25 (19%)	100 (13%)	125 (14%)
Uncertain	4 (3%)	23 (3%)	27 (3%)
			
*Type of surgery*			
Mastectomy	113 (86%)	577 (77%)	690 (78%)
Breast conserving	19 (14%)	176 (23%)	195 (22%)
			
*Tumour classification*			
T1	— (−)	2 (0%)	2 (0%)
T2	30 (23%)	171 (23%)	201 (23%)
T3	78 (59%)	460 (61%)	538 (61%)
Unknown	24 (18%)	120 (16%)	144 (16%)
			
*Number of positive lymph nodes*			
4–9	83 (63%)	485 (64%)	568 (64%)
⩾10	49 (37%)	268 (36%)	317 (36%)
			
*Grade*			
I	19 (14%)	135 (18%)	154 (17%)
II	29 (22%)	263 (35%)	292 (33%)
III	35 (27%)	331 (44%)	366 (41%)
Not determined	49 (37%)	24 (3%)	73 (8%)

**Table 3:** Comparison: TOP2A amplification/HER2 overexpression

**TOP2A amplification**			
**HER2 positivity**	**Positive**	**Negative**	**Total**
Positive	47 (22%)	147 (69%)	194
Negative	2 (1%)	17 (8%)	19
Total	49	164	213

HER2=human epithelial receptor type 2; TOP2A=topoisomerase II*α*.

Overview of HER2/*neu* positivity and the amplification of topoisomerase II*α* in 213 patients. HER2 positivity is defined as overexpression (3+ score by IHC) or HER2 gene amplification detected by CISH (performed for IHC 1+ and 2+ cases).

**Table 4:** Frequency of the immunostainings defining the basal-like subtype of invasive breast carcinomas

**Antigen**	**Interpretable staining**	**Positive staining**	**Negative staining**
HER2/neu	826	205 (25%)	621 (75%)
ER	823	577 (70%)	246 (30%)
CK5/6	762	92 (12%)	670 (88%)
EGFR	763	116 (15%)	647 (85%)

CK5/6=cytokeratin 5/6; EGFR=epidermal growth factor receptor; ER=estrogen receptorHER2=human epithelial receptor type 2.

**Table 5:** Frequency of the subtypes of 753 invasive breast cancers defined by immunohistochemistry

**Group**	**HER2/neu**	**ER**	**CK5/6 and/or EGFR**	**Frequency**
HER2	Positive	Any	Any	205 (27%)
ER (luminal)	Negative	Positive	Negative	411 (55%)
Basal-like	Negative	Negative	One or both positive	98 (13%)
Negative	Negative	Negative	Negative	39 (5%)

CK5/6=cytokeratin 5/6; EGFR=epidermal growth factor receptor; ER=estrogen receptor; HER2=human epithelial receptor type 2.
